# Comparative Indoor Pollution from Glo, Iqos, and Juul, Using Traditional Combustion Cigarettes as Benchmark: Evidence from the Randomized SUR-VAPES AIR Trial

**DOI:** 10.3390/ijerph17176029

**Published:** 2020-08-19

**Authors:** Mariangela Peruzzi, Elena Cavarretta, Giacomo Frati, Roberto Carnevale, Fabio Miraldi, Giuseppe Biondi-Zoccai, Sebastiano Sciarretta, Francesco Versaci, Vittoria Cammalleri, Pasquale Avino, Carmela Protano, Matteo Vitali

**Affiliations:** 1 Department of Medico-Surgical Sciences and Biotechnologies, Sapienza University of Rome, Corso della Repubblica 74, 04100 Latina, Italy; elena.cavarretta@uniroma1.it (E.C.); giacomo.frati@uniroma1.it (G.F.); roberto.carnevale@uniroma1.it (R.C.); giuseppe.biondizoccai@uniroma1.it (G.B.-Z.); sebastiano.sciarretta@uniroma1.it (S.S.); 2 Mediterranea Cardiocentro, 80122 Naples, Italy; 3 IRCCS NEUROMED, 86077 Pozzilli, Italy; 4 Department of Clinical, Internal Medicine, Anesthesiology and Cardiovascular Sciences, Sapienza University of Rome, Viale Del Policlinico 155, 00161 Rome, Italy; fabio.miraldi@uniroma1.it; 5 UOC UTIC Emodinamica e Cardiologia, Ospedale Santa Maria Goretti, Via Antonio Canova, 04100 Latina, Italy; francescoversaci@yahoo.it; 6 Department of Public Health and Infectious Diseases, Sapienza University of Rome, 00185 Rome, Italy; vittoria.cammalleri@uniroma1.it (V.C.); carmela.protano@uniroma1.it (C.P.); matteo.vitali@uniroma1.it (M.V.); 7 Department of Agricultural, Environmental and Food Sciences (DiAAA), University of Molise, via De Sanctis, 86100 Campobasso, Italy; avino@unimol.it

**Keywords:** Glo, indoor, Iqos, Juul, pollution, reduced risk product, passive smoking, smoking

## Abstract

Modified risk products (MRP) such as electronic vaping cigarettes (EVC) and heat-not-burn cigarettes (HNBC) are appealing alternatives to combustion cigarettes. Limited between- and within-device comparative data are available on MRP. We aimed at comparing indoor particulate matter (PM) emissions measured in a randomized trial enforcing standardized smoking sessions, testing different devices and flavors of MRP, using traditional combustion cigarettes (TCC) as benchmark. Overall, MRP yielded significantly lower levels of indoor PM in comparison to TCC (with median PM levels during smoking for MRP < 100 μg/m^3^, and for TCC > 1000 μg/m^3^). Despite this, significant differences among MRP were found, with Iqos appearing associated with a significantly lower burden of emissions for all the monitored fractions of PM, including total PM (all *p* < 0.05). Precisely, during use, PM ≤1 µm (PM_1_) emissions were 28 (16; 28) μg/m^3^ for Glo, 25 (15; 57) μg/m^3^ for Iqos, and 73 (15; 559) μg/m^3^ for Juul (*p* < 0.001 for Glo vs. Iqos, *p* < 0.001 for Glo vs. Juul, and *p* = 0.045 for Iqos vs. Juul). Exploratory within-MRP analyses suggested significant differences between flavors, favoring, for instance, Ultramarine for Glo, Bronze for Iqos, and Mango for Juul, even if results varied substantially according to individual smoker. In conclusion, leading MRP have significantly less intense and persistent effects on indoor pollution in comparison to TCC. Yet, when focusing solely on MRP, between-product and between-flavor differences appear, with quantitative estimates suggesting lower polluting effects with Iqos. These results, if confirmed externally, could be used to individualize product and flavor choice to minimize the untoward effects of EVC and HNBC on indoor pollution.

## 1. Introduction

Smoking of traditional combustion cigarettes (TCC) is associated with major burdens of mortality, morbidity, and cost [1,2]. Recently, novel smoking approaches have been introduced, with very favorable market penetration, including electronic vaping cigarettes (EVC) and heat-not-burn cigarettes (HNBC) [3,4]. The popularity of EVC has considerably increased during the past decades in the United States [5] and in European countries [6]. Similarly, the use of HNBC has rapidly increased worldwide, particularly among young individuals [7]. Whether these novel modified risk products (MRP) may actually prove as reduced risk products (RRP) remains still uncertain, despite several reports suggesting that both EVC and HNBC may prove less detrimental than TCC in several dimensions [8,9].

Indeed, by minimizing combustion (HNBC) or avoiding it altogether (EVC), MRP may profoundly reduce the risks associated with smoking, for instance, by reducing emissions of particulate matter (PM) and carbon monoxide (CO) [10,11,12,13,14], despite still delivering nicotine, with its established detrimental clinical effects [15]. Intriguingly, while EVC would theoretically appear less toxic than HNBC given the vaporization methods, the possible presence of volatile toxic agents may undermine such purported benefits [10,16]. This might hold even truer when specific flavors are used, given the potential to increase exponentially the release of potentially hazardous agents, putting particularly some individuals at risk who could be more susceptible than others [17]. Accordingly, several reports have highlighted that MRP cannot be considered risk-free, given the multidimensional hazards associated with their use, chronic as well as acute [18,19,20].

Despite an expanding evidence base on the reduced risk of indoor pollution and ensuing passive smoking associated with MRP, comparative analyses focusing on different MRP are limited in scope and detail, as well as methodology [16,21,22]. In particular, most studies focusing on MRP published to date had one or more of the following methodological weaknesses: being non-randomized, lacking comprehensive panels of different MRP, or lacking state-of-the-art multilevel statistical models for data analysis. Moreover, few if any reports have compared MRP at large, and then, focused on MRP flavors, which may impact on short and long-term safety [23]. Recently, we have described results of a randomized trial which showed significantly lower indoor emissions of PM when using Glo (British American Tobacco, London, UK), or Iqos (Altria, Richmond, VA, USA) as HNBC and Juul (Juul Labs, San Francisco, CA, USA) as EVC, in comparison to TCC [24]. PM, a well-known toxic and carcinogen independently from its physical status (solid or liquid) and chemical composition [25], was used in such study as a global indicator of indoor air pollution. We hereby aim at providing a more poignant comparison of aggregate MRP, as well as different flavors of each MRP type, in order to expand and confirm prior findings, while capitalizing on modern state-of-the-art statistical modeling tools [26,27].

## 2. Materials and Methods

### 2.1. Design and Experiment

Details of the methodology of the SUR-VAPES AIR (Sapienza University of Rome-Vascular Assessment of Proatherosclerotic Effects of Smoking Ambient Indoor) randomized trial have been provided elsewhere [24], including the institutional funding and ethical approval (Sapienza University of Rome Identifier 3520). Briefly, after generating a randomization list with blocking based on MRP type, 7 current smokers were assigned one of the products to smoke in standardized conditions according to a 2-block set of 15 sessions each, for a total of 30 sessions (thus, yielding 15 device/flavoring combinations repeated twice). The 7 subjects recruited for the experiments were smokers of traditional cigarettes that converted to dual smoking (e.g., both TCC smokers and MRP users). The combinations of device/flavoring resulting from the randomization were as follows Glo2 (Glo with Neo Beryl), Iqos3 (Iqos with Heets Bronze), Glo4 (Glo with Neo Yellow), Iqos6 (Iqos with Heets Yellow), Iqos1 (Iqos with Heets Amber), Glo3 (Glo with Neo Ultramarine), Juul4 (Juul with Royal Creme), Juul3 (Juul with Mint), Juul2 (Juul with Mango), Iqos2 (Iqos with Heets Blue), Iqos5 (Iqos with Heets Turquoise), Marlboro Gold (Philip Morris International, Richmond, VA, USA, for TCC), Juul1 (Juul with Golden Tobacco), Glo1 (Glo with Neo Aegean), Iqos4 (Iqos with Heets Sienna), Iqos2 (Iqos with Heets Blue), Marlboro Gold (Philip Morris International, Richmond, VA, USA, for TCC), Iqos3 (Iqos with Bronze), Glo1 (Glo with Neo Aegean), Iqos5 (Iqos with Heets Turquoise), Juul2 (Juul with Mango), Juul4 (Juul with Royal Creme), Iqos1 (Iqos with Heets Amber), Iqos4 (Iqos with Heets Sienna), Glo2 (Glo with Beryl), Glo4 (Glo with Neo Yellow), Iqos6 (Iqos with Heets Yellow), Juul1 (Juul with Golden Tobacco), Juul3 (Juul with Mint), and Glo3 (Glo with Neo Ultramarine). Glo2 was smoked by smoker 1, Iqos3 by smoker 2, Glo4 by smoker 3, Iqos6 by smoker 4, Iqos1 by smoker 5, Glo3 by smoker 6, Juul4 by smoker 7, and so on. Notably, part of the experiments hereby reported have already been described in Protano et al., where, however, only data from 3 smokers were included [24]. In particular, the chosen TCC is characterized by a mean content/yield of 0.5 mg nicotine, 6 mg tar, and 7 mg carbon monoxide per cigarette according to Braun et al. [28].

Emissions of PM with diameter ≤10 µm (PM_10_), ≤4 µm (PM_4_), ≤2.5 µm (PM_2.5_), and ≤1 µm (PM_1_) were continuously measured in real use conditions 5 min before, during, and 5 min after smoking each product in a room of 53 m^3^, with temperature and relative humidity ranging between 20 and 23 °C and 36 and 40%, respectively (Figure S1). The air exchange rate (λ = 0.69 h^−1^) of the test room was calculated using the CO_2_ tracer gas technique, as previously reported [24,29,30,31]. During each experiment, windows and doors were maintained closed. Conversely, they were opened after each experiment until room conditions were again at initial levels of PM. The aerosol concentrations (μg/m^3^) for PM_10_, PM_4_, PM_2.5_, and PM_1_ were measured with 3 s time resolution, using a portable, laser-operated aerosol mass analyzer (Dusttrak II Aerosol Monitor, model 8530, TSI, 0.1–10 µm particle size range, TSI, Shoreview, MN, USA). The measurements were carried out in “cumulative” mode, including the mass of all particles smaller than or equal to the defined size. The aerosol was sampled directly through the entry of the instrument without using any tube or collector. The instrument was placed approximately 1.5 m above the floor level and approximately 1.5 m from the smoker, accruing hundreds of measurements for each smoking session (Figure S1). Twelve puffs were made for each session that lasted about 5.5 min (1 puff each thirty seconds), since the common way of smoking typically consists of 10–12 puffs of a cigarette, for a period of about 5–6 min [24].

### 2.2. Analysis

Descriptive analysis was based on median, 1st quartile, and 3rd quartile. Inferential analysis was based, for exploratory purposes, on a mixed linear model with Gaussian likelihood using as fixed effects, timing (before, during, or after smoking) and session identifier, and as random effects, subject identifier. Specifically, this analytical approach expands, in terms of scope and precision, the previous findings as reported by Protano et al. [24]. Exploiting such a refined analytical framework, which takes into account all measurements, recognizing in the multilevel model the individual, the session, the phase (before, during and after smoking), the MRP subtype, and the MRP type (and thus, capable of simultaneously capturing within-subject, within-session, within-MRP subtype, and within-MRP type effects, as well as between-subject, between-session, between-MRP subtype, and between-MRP type effects), we focused first on the comparison between Glo, Iqos, and Juul, using TCC as the benchmark, after log10 transformation. Then, we performed within-MRP comparisons of flavors (e.g., comparing the 6 different flavors of Iqos). Finally, we explored between-subject variability in PM emissions. Statistical significance was set at the 2-tailed 0.05 level, without multiplicity adjustments. Computations and visualizations were performed with Stata 13 (StataCorp, College Station, TX, USA) with the meglm package, and R 3.6.3 (R Foundation for Statistical Computing, Vienna, Austria) with the ggplot2 package.

## 3. Result

Overall, MRP yielded significantly lower levels of indoor PM in comparison to TCC (Table 1). In particular, during smoking sessions, median PM levels rose to >1000 μg/m^3^, with further increases due to exhalation and redistribution in the after smoking phases, at odds with median PM levels always lower than 100 μg/m^3^ with MRP, irrespective of the flavor or smoking phase (Table 1, Table 2, Table 3 and Table 4; Table S1).

**Figure 1:**
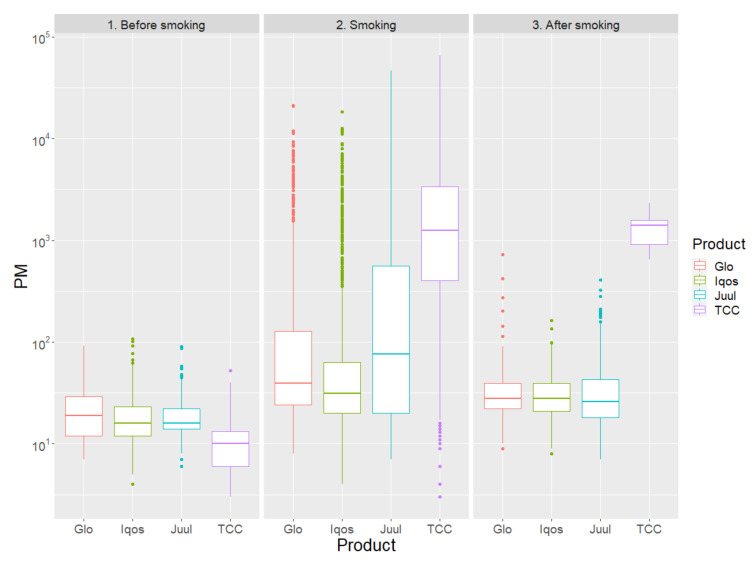
Total particulate matter (PM) concentrations, expressed as μg/m^3^, comparing Glo, Iqos, Juul, and traditional combustion cigarettes (TCC), distinguishing the following phases: before smoking (1), smoking (2), and after smoking (3).

Notwithstanding the limited overall indoor contamination associated with MRP in comparison to TCC, between- and within-MRP comparative analysis showed significant differences in PM emissions (Table 1, Figure 1).

Specifically, Iqos appeared associated with a significantly lower burden of emissions for all classes of PM, including total PM emissions (all *p* < 0.05), even if differences were of relatively small magnitude, and substantial variability and skewness were evident. In addition, PM concentrations quickly decreased after use with all MRP. Precisely, total PM concentrations measured in the test room during use were 39 (24; 127) μg/m^3^ for Glo, 31 (20; 63) μg/m^3^ for Iqos, and 76 (20; 565) μg/m^3^ for Juul (*p* < 0.001 for Glo vs. Iqos, *p* < 0.001 for Glo vs. Juul, and *p* = 0.021 for Iqos vs. Juul), actually profiling Iqos as less polluting and Glo as more polluting. Similar effects were found for PM_10_, PM_4_, PM_2.5_, and PM_1_ (all *p* < 0.05).

**Figure 2:**
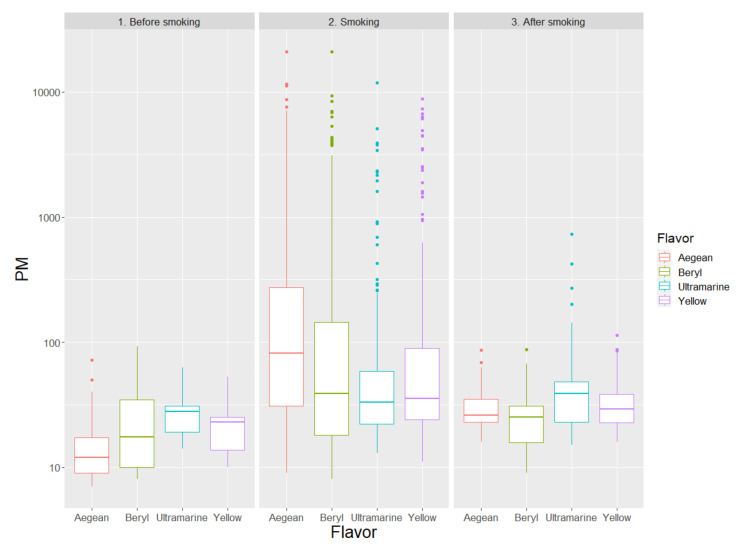
Levels of total particulate matter (PM), expressed as μg/m^3^, comparing different flavors of Glo, distinguishing the following phases: before smoking (1), smoking (2), and after smoking (3).

Within-MRP comparisons provided evidence that flavorings may impact PM indoor emissions, either because of smoke features or because of different smoking patterns (e.g., puff frequency and depth, or nasal vs. oral expiration), for all types of MRP under investigation. In particular, different Glo flavors were associated with significant differences in PM emissions (Table 2, Figure 2), with Ultramarine being associated with the lowest levels of PM of any size, at odds, for instance, with Aegean (e.g., for PM_10_ concentrations during use, which were, respectively, 33 (22; 59) vs. 82 (31; 277) μg/m^3^, *p* = 0.027).

**Figure 3:**
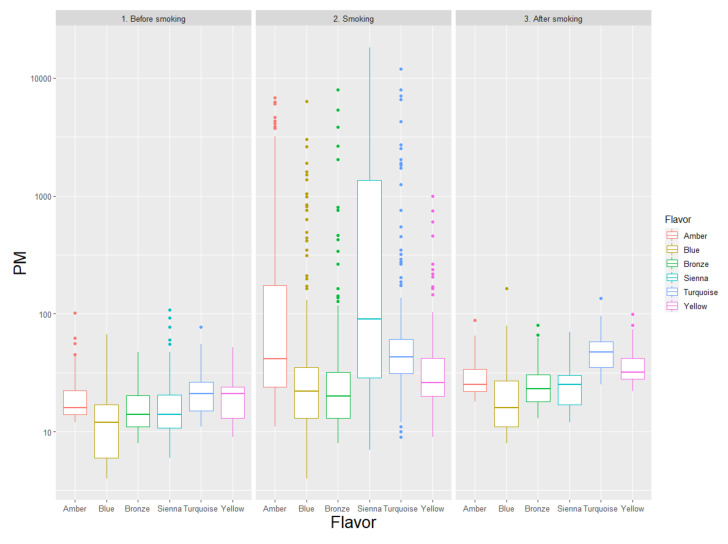
Levels of total particulate matter (PM), expressed as μg/m^3^, comparing different flavors of Iqos, distinguishing the following phases: before smoking (1), smoking (2), and after smoking (3).

Similar analyses were conducted for Iqos (Table 3, Figure 3), highlighting that the Bronze flavor was associated with the lowest PM emissions, at odds, for instance, with Sienna, which yielded the highest concentrations of indoor PM (e.g., for PM_2.5_ concentrations during use, which were, respectively, 14 (11; 25) vs. 79 (22; 1370) μg/m^3^, *p* < 0.001).

**Figure 4:**
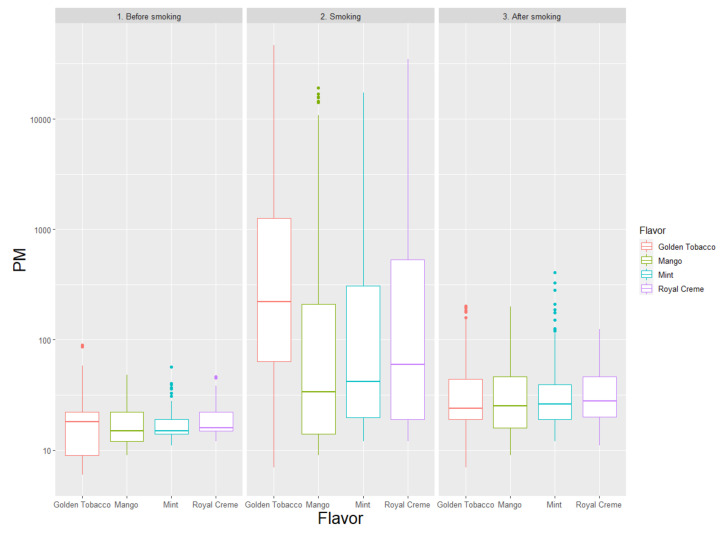
Levels of total particulate matter (PM), expressed as μg/m^3^, comparing different flavors of Juul, distinguishing the following phases: before smoking (1), smoking (2), and after smoking (3).

Even for Juul, between-flavor differences appeared significant, at least in terms of nominal statistical thresholds (Table 4, Figure 4). In particular, across the four flavors tested, Mango was associated with the lowest emissions of PM, at odds in particular with Golden Tobacco, which appeared associated with almost twice larger emissions (e.g., PM_4_ levels during use were, respectively, 17 (11; 199) vs. 214 (61; 1270) μg/m^3^, *p* = 0.042).

Finally, exploratory analysis for between-smoker differences highlighted significant differences and variability in patterns of emissions, with some smokers generating lower PM concentrations, and others, higher PM concentrations, in some cases, with significant variability and skewness (Table S1, *p* < 0.001).

## 4. Discussion

The present work, building upon a randomized trial comparing different leading MRP and focusing on indoor pollution in a standardized experimental setting, has the following major implications. First, the MRP under investigation in the present study, i.e., Glo, Iqos, and Juul, are associated with trivial increases in indoor pollution in comparison to TCC. Second, notwithstanding the limited impact of indoor MRP use on indoor PM concentrations, statistically significant differences between Glo, Iqos, and Juul in terms of indoor pollution appear evident, with Iqos appearing less polluting and Glo more polluting, at least in relative terms. Third, within each MRP type, flavor may impact on polluting effects, either because of smoke features or because of indirect effects mediated by smoking patterns (e.g., nasal vs. oral expiration). Fourth, there remains substantial individual variability, such that indoor pollution may be high when MRP are used by some smokers, and low when used by others. Fifth, we cannot provide a consistent explanation concerning between-flavor variability such as in the case of the less polluting effects of Iqos Bronze. We can speculate that, as in the case of TCC, menthol, by having local anesthetic properties, could conceal the negative sensations of smoking, due to desensitizing receptors [32]. Accordingly, smokers could hold their breath more and thus, reduce emissions during expiration. Irrespective of these potential confounding effects, our findings have important implications in the sense that may help, if confirmed externally in larger series, smokers wishing to use MRP as a risk reduction strategy (i.e., to quit altogether TCC), to pick the one which is less likely to be harmful, at least in terms of indoor pollution [33].

The evidence base on MRP is expanding exponentially, and it is clear that, despite ongoing efforts at regulating their use (e.g., with increased taxation), MRP usage will continue to grow [3,8,9,34]. Accordingly, it is paramount to expand the evidence concerning these products, with details worth being sought ranging from cardiovascular effects to additivity and polluting impact. Indeed, while the dramatic and persisting indoor polluting effect of TCC is very clear, MRP also adversely modify indoor ambient air [20], for instance, by releasing PM and other established or potentially toxic agents [35]. This holds true for HNBC as well as EVC, despite their evident inherent differences [35,36]. In particular, it is established that even single usage of MRP increases indoor PM levels significantly [36]. Our work builds upon such findings and provides, to date, the first thorough comparison between two leading HNBC, Glo and Iqos, with a leading EVC, Juul. Interestingly, MRP appear unequal in terms of indoor polluting effects, in the sense that in our study, Iqos was associated with less indoor polluting effects than Glo and, albeit to a lesser extent, Juul. Accordingly, indoor pollution associated with MRP is not MRP-type specific, but rather, device-specific.

Furthermore, building upon accruing evidence suggesting the impact of different flavors of a given MRP on its risk profile, we explored within-device (i.e., between-flavor) polluting effects, finding indeed that some flavors are associated with more indoor pollution than others [37,38,39,40,41,42,43,44,45]. Notwithstanding the evident impact of smoker features (which may range from habitus to cardiopulmonary physiology or smoking style), it is clear that MRP should not be considered all identical, and even specific flavors may be more or less hazardous than others [38,41]. Indeed, differences in exhaled aerosols from different smokers depend on many factors, including individual characteristics (i.e., age, gender, lung capacity) and status (stress, anxiety, time since the last cigarette, nicotine addiction degree, etc.), in addition to the specific way of smoking (puff frequency, intensity, volume, and duration at different stages of cigarette consumption, as well as breath hold) [46]. Thus, different smokers/vapers generated different shapes of exhaled aerosol, but this is a common limitation of all experiments performed by humans. Despite these interesting results, further research is required to expand our findings. In particular, a larger sample is required for external validation. In addition, other dimensions of toxicity should be appraised (e.g., aromatic cyclic compounds or heavy metals). Similarly, mitigating factors will need careful appraisal, such as ambient volume, impact of aeration, and so forth. Finally, the association between passive and active smoking effects remains the focus of intense research [47], especially when considering also other established and cardiovascular risk factors [48].

An important issue of our work is the applicability of our findings on Iqos to other HNBC, and, similarly, on Juul to other EVC. In fact, other HNBC devices are available in selected markets, with underlying mechanisms to heat tobacco that differ, at least in part, from Glo and Iqos. Given the evident differences between Glo and Iqos hereby described, we can expect that PM emissions may be specific to each HNBC type, and even its flavors. Similar arguments may apply to EVC. Indeed, we chose Juul for this trial because, on top of being an established market player, it is characterized by default standardization of aerosol emissions. Many EVC can, however, let users customize exposure, in terms of both solution components, concentration, and volume. Accordingly, we cannot safely recommend extrapolating our results on Juul towards other EVC. Accordingly, most likely some EVC may prove significantly more harmful than Juul in terms of toxic emissions [49,50].

This work has several drawbacks, which range from the small sample size to the focus on only three MRP. In addition, apparently healthy volunteers participated in the trial, and thus, our results cannot be considered immediately applicable to patients with cardiopulmonary disease. Notably, differences in indoor polluting effects, while often statistically significant, were of modest magnitude between- and within-MRP, especially when compared to the much greater impact of TCC, and thus, their clinical impact remains to be determined [51]. In addition, we focused our measurements only on PM, but the potential scope of toxic agents released by MRP is very wide, and thus, additional studies with a multidimensional measurement scope are required. Moreover, in vitro experimental studies should be performed to assess whether the observed indoor PM concentrations could increase inflammation and oxidative stress. Furthermore, it is evident that Juul mostly emits liquid droplets, whereas PM produced by HNBC consists of largely solid material. Finally, different analytical approaches can yield different comparative and inferential estimates, given the clear impact of focusing only on smoking sessions vs. more comprehensive pre-, during- and post-smoking sessions, as clearly showed by the differences between the present work and Protano et al. [24]. Yet, it remains undisputed that MRP cannot be considered equal in terms of PM indoor emissions, in terms of both between- and within-MRP comparisons.

## 5. Conclusions

Leading MRP such as Glo, Iqos, and Juul have significantly less intense and persistent effects on indoor pollution in comparison to TCC. Yet, when focusing solely on MRP, between-product and between-flavor differences appear, with quantitative estimates suggesting lower polluting effects with Iqos. These results, if confirmed externally, could be used to individualize product and flavor choice to minimize the untoward effects of EVC and HNBC.

## Figures and Tables

**Table 1:** Levels of particulate matter (PM), expressed as μg/m^3^, comparing Glo, Iqos, Juul, and traditional combustion cigarettes (TCC) *.

Device	Timing	PM_10_	PM_4_	PM_2.5_	PM_1_	Total
**Glo**	Before	16 (11; 24)	14 (10; 23)	14 (10; 23)	14 (10; 22)	19 (12; 29)
	During	31 (19; 120)	29 (17; 120)	28 (17; 120)	28 (16; 28)	39 (24; 127)
	After	25 (19; 31)	25 (18; 29)	24 (18; 29)	24 (18; 29)	28 (22; 39)
**Iqos**	Before	14 (11; 19)	13 (10; 17)	13 (10; 17)	13 (10; 17)	16 (12; 23)
	During	26 (17; 59)	25 (16; 57)	25 (15; 57)	25 (15; 57)	31 (20; 63)
	After	25 (19; 31)	25 (17; 30)	24 (17; 30)	24 (17; 29)	28 (21; 39)
	Before	15 (13; 18)	14 (12; 17)	14 (12; 16)	14 (12; 16)	16 (14; 22)
**Juul**	During	75 (17; 565)	73 (16; 565)	73 (15; 565)	73 (15; 559)	76 (20; 565)
	After	23 (16; 35)	22 (14; 34)	21 (14; 33)	21 (14; 33)	26 (18; 43)
	Before	10 (6; 12)	9 (5; 11)	8 (4; 11)	8 (4; 11)	10 (6; 14)
**TCC**	During	1245 (392; 3360)	1245 (392; 3360)	1245 (391; 3360)	1245 (391; 3350)	1250 (401; 3360)
	After	1400 (896; 1580)	1390 (894; 1580)	1390 (894; 1580)	1390 (892; 1580)	1400 (905; 1580)
**P Glo vs. Iqos**	-	<0.001	<0.001	<0.001	<0.001	<0.001
**P Glo vs. Juul**	-	<0.001	<0.001	<0.001	<0.001	<0.001
**P Glo vs. TCC**	-	<0.001	<0.001	<0.001	<0.001	<0.001
**P Iqos vs. Juul**	-	0.016	0.031	0.038	0.045	0.021
**P Iqos vs. TCC**	-	<0.001	<0.001	<0.001	<0.001	<0.001
**P Juul vs. TCC**	-	<0.001	<0.001	<0.001	<0.001	<0.001

* Reported as median (1st quartile; 3rd quartile); this analysis is based on a multilevel model assuming a homogenous within-modified risk product type effect, thus, comparing all measurements from Glo with all measurements from Iqos and all measurements from Juul, taking into account, however, smoker, session, and timing; PM_10_ = PM with diameter ≤ 10 µm; PM_4_ = PM with diameter ≤ 4 µm; PM_2.5_ = PM with diameter ≤ 2.5 µm; PM_1_ = PM with diameter ≤ 1 µm.

**Table 2:** Levels of particulate matter (PM), expressed as μg/m^3^, comparing different flavors of Glo *.

Flavor	Timing	PM_10_	PM_4_	PM_2.5_	PM_1_	Total
**Aegean**	Before	12 (9; 18)	10 (8; 11)	10 (8; 11)	10 (8; 12)	11 (9; 13)
	During	82 (31; 277)	75 (22; 276)	75 (22; 276)	75 (22; 276)	76 (24; 277)
	After	26 (23; 35)	24 (21; 28)	24 (21; 28)	24 (21; 28)	25 (22; 29)
**Beryl**	Before	18 (10; 35)	13 (8; 17)	13 (9; 18)	13 (9; 18)	15 (10; 20)
	During	39 (18; 145)	28 (13; 128)	28 (14; 130)	28 (14; 130)	30 (15; 136)
	After	25 (16; 31)	23 (12; 25)	23 (13; 26)	23 (13; 26)	24 (14; 27)
**Ultramarine**	Before	28 (19; 31)	23 (14; 27)	24 (14; 27)	24 (15; 28)	26 (16; 29)
	During	33 (22; 59)	25 (17; 32)	26 (17; 33)	26 (17; 33)	28 (19; 37)
	After	39 (23; 48)	27 (18; 38)	28 (18; 39)	28 (19; 39)	34 (20; 42)
	Before	23 (14; 25)	22 (12; 23)	22 (12; 24)	22 (13; 24)	13 (22; 24)
**Yellow**	During	36 (24; 90)	26 (22; 82)	27 (22; 82)	27 (23; 82)	29 (24; 83)
	After	29 (23; 39)	27 (17; 29)	27 (18; 29)	27 (18; 30)	28 (20; 31)
**P Aegean vs. Beryl**	-	0.001	<0.001	<0.001	<0.001	<0.001
**P Aegean vs. Ultramarine**	-	0.027	0.013	0.010	0.007	0.126
**P Aegean vs. Yellow**	-	0.049	0.041	0.036	0.029	0.114
**P Beryl vs. Ultramarine**	-	0.132	0.168	0.168	0.162	0.125
**P Beryl vs. Yellow**	-	0.031	0.020	0.017	0.014	0.099
**P Ultramarine vs. Yellow**	-	0.658	0.482	0.457	0.416	0.975

* Reported as median (1st quartile; 3rd quartile); PM_10_ = PM with diameter ≤ 10 µm; PM_4_ = PM with diameter ≤ 4 µm; PM_2.5_ = PM with diameter ≤ 2.5 µm; PM_1_ = PM with diameter ≤ 1 µm.

**Table 3:** Levels of particulate matter (PM), expressed as μg/m^3^, comparing different flavors of Iqos *.

	Measurements	PM_10_	PM_4_	PM_2.5_	PM_1_	Total
	Before	15 (14; 17)	14 (14; 16)	14 (14; 16)	14 (13; 15)	16 (14; 23)
**Amber**	During	32 (24; 172)	31 (22; 170)	30 (22; 170)	30 (22; 170)	42 (24; 176)
	After	24 (22; 27)	23 (21; 26)	23 (21; 26)	23 (20; 25)	25 (22; 34)
	Before	11 (6; 13)	11 (5; 13)	11 (5; 13)	10 (5; 13)	12 (6; 17)
**Blue**	During	19 (11; 27)	17 (10; 26)	10 (7; 26)	17 (10; 26)	22 (13; 35)
	After	14 (10; 21)	12 (9; 19)	12 (9; 19)	12 (9; 19)	16 (11; 27)
	Before	12 (10; 15)	11 (9; 14)	10 (9; 13)	10 (9; 13)	14 (11; 21)
**Bronze**	During	16 (12; 26)	14 (11; 25)	14 (11; 25)	14 (11; 25)	20 (13; 32)
	After	20 (17; 25)	18 (16; 24)	18 (15; 24)	18 (15; 23)	23 (18; 31)
	Before	13 (9; 16)	12 (8; 14)	12 (7; 14)	12 (7; 14)	14 (11; 21)
**Sienna**	During	80 (25; 1370)	80 (23; 1370)	79 (22; 1370)	79 (22; 1370)	90 (28; 1370)
	After	23 (17; 27)	22 (15; 26)	22 (15; 25)	22 (15; 25)	25 (17; 30)
	Before	21 (15; 25)	20 (14; 23)	19 (14; 23)	19 (14; 22)	21 (15; 27)
**Turquoise**	During	39 (26; 51)	37 (24; 49)	37 (24; 49)	37 (24; 49)	43 (31; 61)
	After	44 (34; 51)	43 (33; 49)	42 (32; 49)	42 (32; 49)	47 (35; 58)
	Before	20 (12; 22)	19 (11; 22)	19 (10; 21)	19 (10; 21)	21 (13; 24)
**Yellow**	During	24 (20; 33)	23 (19; 32)	22 (19; 32)	22 (19; 32)	26 (20; 42)
	After	30 (28; 35)	29 (27; 32)	29 (27; 32)	28 (26; 32)	32 (28; 42)
**P Amber vs. Blue**	-	<0.001	<0.001	<0.001	<0.001	<0.001
**P Amber vs. Bronze**	-	0.002	0.003	0.003	0.004	<0.001
**P Amber vs. Sienna**	-	0.122	0.175	0.190	0.202	0.078
**P Amber vs. Turquoise**	-	0.637	0.725	0.743	0.751	0.581
**P Amber vs. Yellow**	-	0.102	0.120	0.125	0.133	0.032
**P Blue vs. Bronze**	-	0.373	0.353	<0.001	0.366	0.403
**P Blue vs. Sienna**	-	<0.001	<0.001	0.353	<0.001	<0.001
**P Blue vs. Turquoise**	-	<0.001	<0.001	<0.001	<0.001	<0.001
**P Blue vs. Yellow**	-	0.020	0.019	<0.001	0.022	0.027
**P Bronze vs. Sienna**	-	<0.001	<0.001	<0.001	<0.001	<0.001
**P Bronze vs. Turquoise**	-	<0.001	<0.001	<0.001	<0.001	<0.001
**P Bronze vs. Yellow**	-	0.001	0.001	0.001	0.001	0.006
**P Sienna vs. Turquoise**	-	0.247	0.279	0.290	0.301	0.195
**P Sienna vs. Yellow**	-	0.001	0.003	0.003	0.004	<0.001
**P Turquoise vs. Yellow**	-	<0.001	<0.001	0.001	0.001	<0.001

* Reported as median (1st quartile; 3rd quartile); PM_10_ = PM with diameter ≤ 10 µm; PM_4_ = PM with diameter ≤ 4 µm; PM_2.5_ = PM with diameter ≤ 2.5 µm; PM_1_ = PM with diameter ≤ 1 µm.

**Table 4:** Levels of particulate matter (PM), expressed as μg/m^3^, comparing different flavors of Juul *.

	Measurements	PM_10_	PM_4_	PM_2.5_	PM_1_	Total
	Before	17 (9; 19)	17 (8; 19)	16 (8; 18)	16 (8; 18)	18 (9; 22)
**Golden Tobacco**	During	216 (61; 1280)	214 (61; 1270)	214 (61; 1270)	214 (60; 1270)	221 (63; 1280)
	After	22 (13; 36)	22 (12; 34)	21 (12; 34)	21 (11; 34)	24 (19; 44)
	Before	15 (12; 17)	14 (11; 16)	14 (11; 15)	13 (10; 15)	15 (12; 22)
**Mango**	During	20 (13; 200)	17 (11; 199)	16 (11; 199)	16 (11; 195)	34 (14; 212)
	After	21 (13; 35)	20 (11; 34)	20 (11; 33)	19 (11; 33)	25 (16; 46)
	Before	15 (14; 17)	14 (14; 15)	14 (13; 15)	14 (13; 15)	15 (14; 19)
**Mint**	During	26 (17; 304)	25 (14; 304)	25 (15; 304)	25 (15; 300)	42 (20; 314)
	After	23 (18; 34)	22 (16; 34)	22 (16; 33)	22 (16; 32)	26 (19; 39)
	Before	15 (14; 17)	15 (13; 16)	15 (13; 16)	14 (13; 16)	16 (15; 23)
**Royal Crème**	During	50 (17; 529)	50 (16; 527)	49 (15; 526)	48 (15; 521)	60 (19; 538)
	After	25 (17; 36)	23 (15; 34)	22 (15; 33)	22 (15; 32)	28 (20; 47)
**P Golden Tobacco vs. Mango**	-	0.055	0.042	0.039	0.035	0.140
**P Golden Tobacco vs. Mint**	-	0.588	0.578	0.570	0.526	0.519
**P Golden Tobacco vs. Royal Crème**	-	0.593	0.608	0.598	0.640	0.712
**P Mango vs. Mint**	-	0.125	0.100	0.096	0.100	0.339
**P Mango vs. Royal Crème**	-	0.008	0.006	0.005	0.005	0.045
**P Mint vs. Royal Crème**	-	0.152	0.155	0.145	0.143	0.177

* Reported as median (1st quartile; 3rd quartile); PM_10_ = PM with diameter ≤ 10 µm; PM_4_ = PM with diameter ≤ 4 µm; PM2.5 = PM with diameter ≤ 2.5 µm; PM_1_ = PM with diameter ≤ 1 µm.

## Supplementary Materials

The following are available online at https://www.mdpi.com/1660-4601/17/17/6029/s1, Figure S1. Example of measurements obtained during a single-modified risk product subtype smoking session by an individual smoker, totalling 262 values. Left panel highlights measurements before smoking, middle panel measurements during smoking, and right panel measurements after smoking, for a total time of 13 min. In the present case, a Glo Aegean was used. PM_10_ = PM with diameter ≤10 µm; PM_4_ = PM with diameter ≤4 µm; PM_2.5_ = PM with diameter ≤2.5 µm; PM_1_ = PM with diameter ≤1 µm., Table S1. Levels of particulate matter (PM), expressed as μg/m3, comparing different smokers.
