# Supplementary material for: Comparative Indoor Pollution from Glo, Iqos, and Juul, Using Traditional Combustion Cigarettes as Benchmark: Evidence from the Randomized SUR-VAPES AIR Trial
Source: Int J Environ Res Public Health. 2020 Aug 19;17(17):6029. doi: 10.3390/ijerph17176029 (PMC7504617; doi:10.3390/ijerph17176029)
Supplement: Supplementary file 1 [file ijerph-17-06029-s001.pdf]

Peruzzi et al, Comparative indoor pollution from Glo, Iqos, and Juul, using traditional combustion cigarettes as benchmark: evidence from the randomized SUR-VAPES AIR trial

Online supplement

Table S1. Levels of particulate matter (PM), expressed as μg/m^3, comparing different smokers.*.

Smoker	PM_{10}	PM_4	$PM_{2.5}$	PM_1	Total
**1**	24 (13; 30)	23 (12; 29)	23 (12; 28)	23 (11; 28)	25 (15; 38)
**2**	28 (23; 35)	27 (22; 34)	27 (21; 33)	27 (21; 33)	29 (24; 42)
**3**	19 (13; 27)	16 (12; 25)	16 (12; 25)	16 (11; 25)	23 (14; 36)
**4**	20 (16; 27)	17 (15; 25)	17 (14; 24)	17 (14; 24)	23 (17; 38)
**5**	29 (19; 100)	27 (18; 98)	27 (17; 98)	27 (17; 97)	35 (22; 106)
**6**	22 (14; 32)	20 (13; 30)	20 (13; 30)	20 (12; 30)	25 (16; 38)
**7**	14 (19; 273)	17 (13; 271)	17 (13; 271)	17 (13; 270)	23 (15; 273)

*reported as median (1st quartile; 3rd quartile); PM_{10} = PM with diameter ≤10 μm; PM_4 = PM with diameter ≤4 μm; $PM_{2.5}$ = PM with diameter ≤2.5 μm; PM_1 = PM with diameter ≤1 μm.

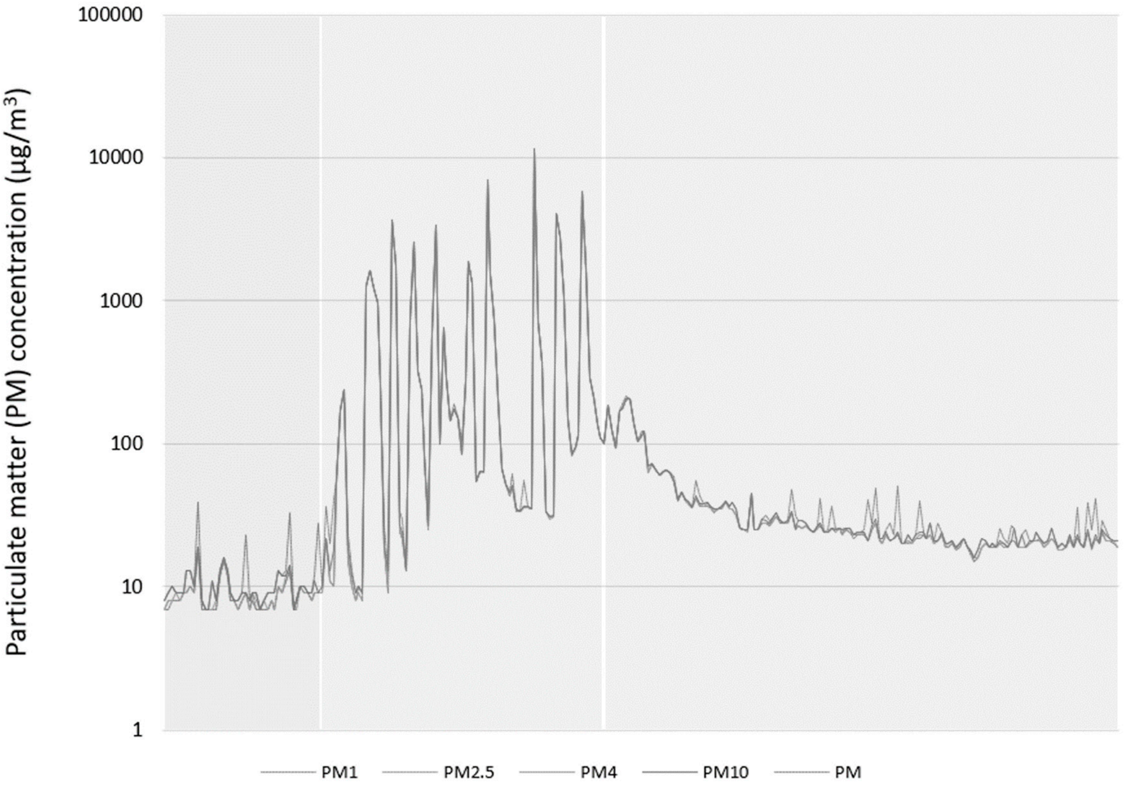

Figure S1. Example of measurements obtained during a single-modified risk product subtype smoking session by an individual smoker, totalling 262 values. Left panel highlights measurements before smoking, middle panel measurements during smoking, and right panel measurements after smoking, for a total time of 13 minutes. In the present case, a Glo Aegean was used. PM10 = PM with diameter ≤10 µm; PM4 = PM with diameter ≤4 µm; PM2.5 = PM with diameter ≤2.5 µm; PM1 = PM with diameter ≤1 µm.
